# Asymmetric Regulation of Peripheral Genes by Two Transcriptional Regulatory Networks

**DOI:** 10.1371/journal.pone.0160459

**Published:** 2016-08-02

**Authors:** Jing-Ru Li, Takahiro Suzuki, Hajime Nishimura, Mami Kishima, Shiori Maeda, Harukazu Suzuki

**Affiliations:** Division of Genomic Technologies, RIKEN Yokohama Institute, 1-7-22 Suehiro-cho, Tsurumi-ku, Yokohama, 230–0045, Kanagawa, Japan; Department of Stem Cell Biology, JAPAN

## Abstract

Transcriptional regulatory network (TRN) reconstitution and deconstruction occur simultaneously during reprogramming; however, it remains unclear how the starting and targeting TRNs regulate the induction and suppression of peripheral genes. Here we analyzed the regulation using direct cell reprogramming from human dermal fibroblasts to monocytes as the platform. We simultaneously deconstructed fibroblastic TRN and reconstituted monocytic TRN; monocytic and fibroblastic gene expression were analyzed in comparison with that of fibroblastic TRN deconstruction only or monocytic TRN reconstitution only. Global gene expression analysis showed cross-regulation of TRNs. Detailed analysis revealed that knocking down fibroblastic TRN positively affected half of the upregulated monocytic genes, indicating that intrinsic fibroblastic TRN interfered with the expression of induced genes. In contrast, reconstitution of monocytic TRN showed neutral effects on the majority of fibroblastic gene downregulation. This study provides an explicit example that demonstrates how two networks together regulate gene expression during cell reprogramming processes and contributes to the elaborate exploration of TRNs.

## Introduction

Gene control in cells is composed of sophisticated functions accomplished by particular transcription factors (TFs) [1]. TFs form a complex transcriptional regulatory network (TRN) to regulate hundreds to thousands of genes, which determines the features of cellular phenotypes such as differentiation, development, and characterized functions [2–7]. The distinctive roles of TFs have been widely applied to cell reprogramming or transdifferentiation; ectopic expression sets of TFs in starting cells successfully induces cell-state changeover including somatic cell reprogramming to the pluripotent state (iPSCs) [8–10], direct conversion between lineages [11–14], and pluripotent stem cell differentiation [15,16].

While cell-fate transition has been widely established, concerns for applications have been raised due to the lack of knowledge of this mechanism [17]. However, the black box of the reprogramming mechanism is gradually becoming uncovered and several functional events crucial for cell reprogramming have been identified [18]. For example, the loss of native characteristics, such as mesenchymal-to-epithelial transition (MET), are necessary in the early phase from fibroblasts to induced pluripotent cells as well as induced dopaminergic neurons [19–21]. A proliferation burst for bypassing apoptosis and switching the metabolic state from oxidative to glycolytic is also required for cell reprogramming from fibroblasts to iPSCs [22,23]. Cell reprogramming is usually achieved by reconstitution of target cell TRN where innate markers are downregulated while target genes are activated. Thus, both start and target cell TRNs simultaneously exist and may interact with each other while cells are being reprogrammed. However, how these TRNs together affect the induction of target cell-specific genes and the downregulation of start cell-specific genes has not been well explored. This is partly due to the limited knowledge of TRNs. We recently determined that PRRX1, OSR1, LHX9, and TWIST2 are core TFs in human dermal fibroblasts, a cell most frequently used as a start cell in cell reprogramming, and showed that mutual regulation among these factors characterizes the specific TRN that safeguards the stability of fibroblasts [24]. In addition, we previously identified core monocytic TFs (SPI1, MNDA, CEBPA, and IRF8) and demonstrated that reconstitution of monocytic TRN by the ectopic expression of four TFs partially reprogrammed fibroblasts into monocyte-like cells [25].

In this study, using transdifferentiation from fibroblasts to monocytes as a platform, we designed a series of parallel experiments of suppression and induction of TRNs to investigate the cross-regulation between intrinsic fibroblastic TRN and imposed monocytic TRN. By comparing the induction of monocytic genes in the presence and absence of fibroblastic TRN suppression, we found that deconstruction of fibroblastic networks significantly improved monocytic gene induction, while reconstituting monocytic TRN did not affect the majority of fibroblastic gene suppression. Collectively, our results indicate the asymmetric regulation of peripheral genes by two TRNs during cell reprogramming.

## Materials and Methods

### Cell culture

Normal human skin fibroblast cells, NB1RGB, were provided by RIKEN Bioresource Center (#RCB0222, Tsukuba, Japan) and were cultured in 10% fetal bovine serum supplemented with minimum essential medium alpha (MEMα, Wako, Japan) at 5% CO_2_ and 37°C. One day after transduction with lentivirus, the medium was changed into the monocyte medium, consisting of RPMI1640 (Wako, Japan) and 20 ng/ml rhM-CSF(Wako, Japan), 20 ng/ml IL-4 (Wako, Japan), 1 mM sodium pyruvate, 50 μM ß-mercaptoethanol, and 10% fetal bovine serum.

### siRNA transfection and RNA isolation

Fibroblast cells at a density of 5 × 10^4^ cells/well were seeded in 6-well culture plates (Nunc, Thermo Scientific, USA) for 24 h. Stealth small interfering RNA (siRNA, 12.5 nM each) or 50 nM of negative control siRNA were pre-mixed with 5 μL Lipofectamine RNAiMAX (Invirtogen, USA) at room temperature for 20 min, followed by transfection. RNA was isolated using NucleoSpin® kit (Macherey-Nagel, Germany) according to the user manual and quantified with NanoDrop (NanoDrop Tech-nologies, USA).

### Plasmid Construction

To overexpress four monocyte TFs at the same time, we tandemly inserted the coding sequence of those genes into one plasmid with the blasticidin resistance gene sequence, followed by lentivirus production (S1 Fig, sequence available on request). Since each gene sequence is spaced by polyA peptide sequences, the four TF genes were first transcribed into one mRNA molecule and translated into one peptide, followed by Peptidase A cleavage and formation into four distinct proteins.

### Overexpression of four TFs using lentivirus transduction

Lentivirus transduction was performed 24 h after siRNA transfection within the 1 μg/ml polybrene-supplemented MEMα at 10 MOI. Fresh monocyte medium (RPMI1640) without polybrene was used 24 h after transduction to avoid cell damage, and 5 μg/ml of blasticidin was added 48 h after the transduction for cell selection.

### Quantitative real-time PCR

All RT-PCR procedures have been previous described [25]. Briefly, the PrimeScript RT-PCR kit (Perfect Real Time, Takara Bi, Japan) and the GeneAMP PCR System 9700 (Applied Biosystems, USA) were used for the reverse transcription reaction. Quantitative real-time PCR (qRT-PCR) was then performed with SYBR Premix EX Taq^TM^ II (Tli RNaseH plus, Takara Bio, Japan) and an ABI 7900 Fast Real-Time PCR System (Applied Biosystems, USA). All reaction conditions and PCR parameters were performed as per manufacturer guidelines. To normalize the expression level of the target mRNA, GADPH mRNA was used as the internal control by using the 2^- ΔΔCT^ method.

### Microarray analysis

Microarray analysis was performed on the Illumina platform. Five hundred nanograms of total RNA were used for first-stranded cDNA and second-stranded cRNA preparation via the Illumina TotalPrep RNA Amplification kit (Ambion, USA). The converted cRNA was then labeled and hybridized using the Human HT-12 v4 Expression BeadChip kit according to the manufacturer’s instruction. Experimental triplicate samples were prepared for microarray analysis. Data were processed with a package from Bioconductor (lumi) [26,27] using a free software environment, R (http://www.r-project.org/). The data had been registered in Gene Expression Omnibus (http://www.ncbi.nlm.nih.gov/geo/) at NCBI (GSE80676).

### Gene set enrichment analysis (GSEA)

We used the java GSEA Desktop Application software from the Broad Institute Gene Set Enrichment Analysis website (http://software.broadinstitute.org/gsea/downloads.jsp). According to the protocol, we created the expression dataset of entire genes (.gct) and selected genes (.gmx). All analyses were run on Java version 8 by 1000 permutation. The phenotype label was set as indicated in the figures.

### Statistical analysis

All data analysis was run on R software environment except for GSEA. We calculated p-value using two-tailed t-test for qPCR results and two-sample Kolmogorov-Smirnov (K-S) test for comparison of fold-change distributions. For microarray and affected genes analysis, the false discovery rates (FDR) were provided using the method of BenjaminiHochberg [26,27]. GSEA provides FDR by computing the ratio of real enrichment score against all permutations dataset and against actual dataset [28].

## Results

### Suppression and induction of two transcriptional regulatory networks

**Fig 1:**
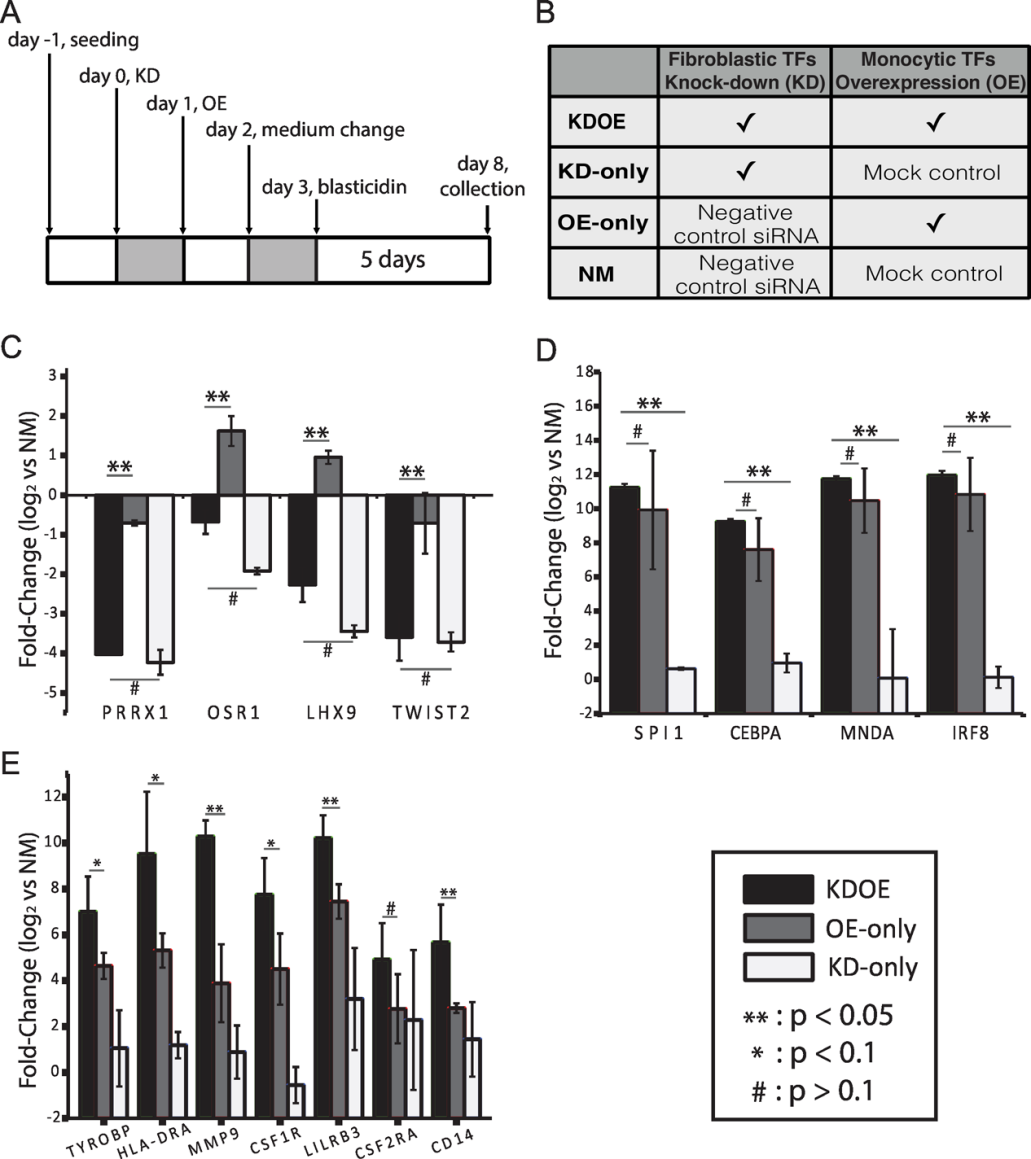
Experimental design and confirmation of knock-down as well as induction of target TF genes. (A) Flow chart of experimental design. Four fibroblastic TFs were suppressed by using siRNA transfection on day 0, and four monocyte TFs were overexpressed on day 1. The monocyte medium was introduced at day 2, followed by sample collection on day 8. (B) Parallel experiments were performed for comparison. (C and D) Validation of four fibroblastic TF knockdown and four monocytic TF induction, respectively. (E) qPCR analysis of monocyte makers revealed increased gene expression levels in KDOE. Independent experiments were repeated a minimum of three times (**: p <0.05, *: p <0.1, #: p >0.1, t-test, black bar: KDOE, gray bar: OE-only, white bar: KD-only).

In order to explore gene regulation of TRNs during the process of transdifferentiation, we designed parallel experiments composed of fibroblastic TRN suppression and monocytic TRN induction. We suppressed fibroblastic TRN by small interfering RNA (siRNA) transfection targeted to four previously identified fibroblastic core TFs (PRRX1, OSR1, LHX9, and TWIST2) [24] and overexpressed four monocytic TFs (SPI1, MNDA, IRF8, and CEBPA) [25] to reconstitute the monocytic TRN. We collected the cells (designated as KDOE) after 5 days of blasticidin selection, where we expected that at this time point, monocytic TRN were under construction (Fig 1A). To clarify the effect of fibroblastic and monocytic TRNs, we performed additional treatments: 1) fibroblasts went through four fibroblastic TF suppression followed by mock-gene lentivirus transduction (designated as KD-only), 2) negative control siRNA transfection and four monocytic TF induction (designated as OE-only), and 3) negative control siRNA transfection and mock-gene transduction (designated as NM, Fig 1B). Suppression and induction of the target TF genes was confirmed by quantitative real-time PCR (qPCR) (Fig 1C and 1D); western blot assays were performed to confirm induction of four monocytic TFs at the protein level (S1B Fig). We observed a similar level of fibroblastic TF suppression between KDOE and KD-only as well as monocytic TF induction between KDOE and OE-only. We further confirmed the induction of representative monocyte marker genes (Fig 1E). Interestingly, we found that KDOE expresses these marker genes significantly higher than both OE-only and KD-only groups, particularly *CD14*, *MMP9*, and *LILRB3*. Of the seven markers checked, only *CSF2RA* was indistinguishably expressed in KDOE and OE-only. These results suggest that KDOE reveals a higher level of monocytic gene expression as a consequence of the regulation in both fibroblastic and monocytic TRNs.

### Cross-regulation of two TRNs

Next, we performed a microarray analysis for entire gene expression. We included fibroblast and monocyte samples in the microarray assay to select cell-specific genes; fibroblastic genes and monocytic genes were selected using stringent criteria of fold-change significance (monocytes vs. fibroblasts, log_2_FC ≤−4, for fibroblastic genes and log_2_FC ≥4, for monocytic genes, both false discovery rates (FDR) <1e-10). A total of 381 monocytic genes and 406 fibroblastic genes were identified under these criteria.

**Fig 2:**
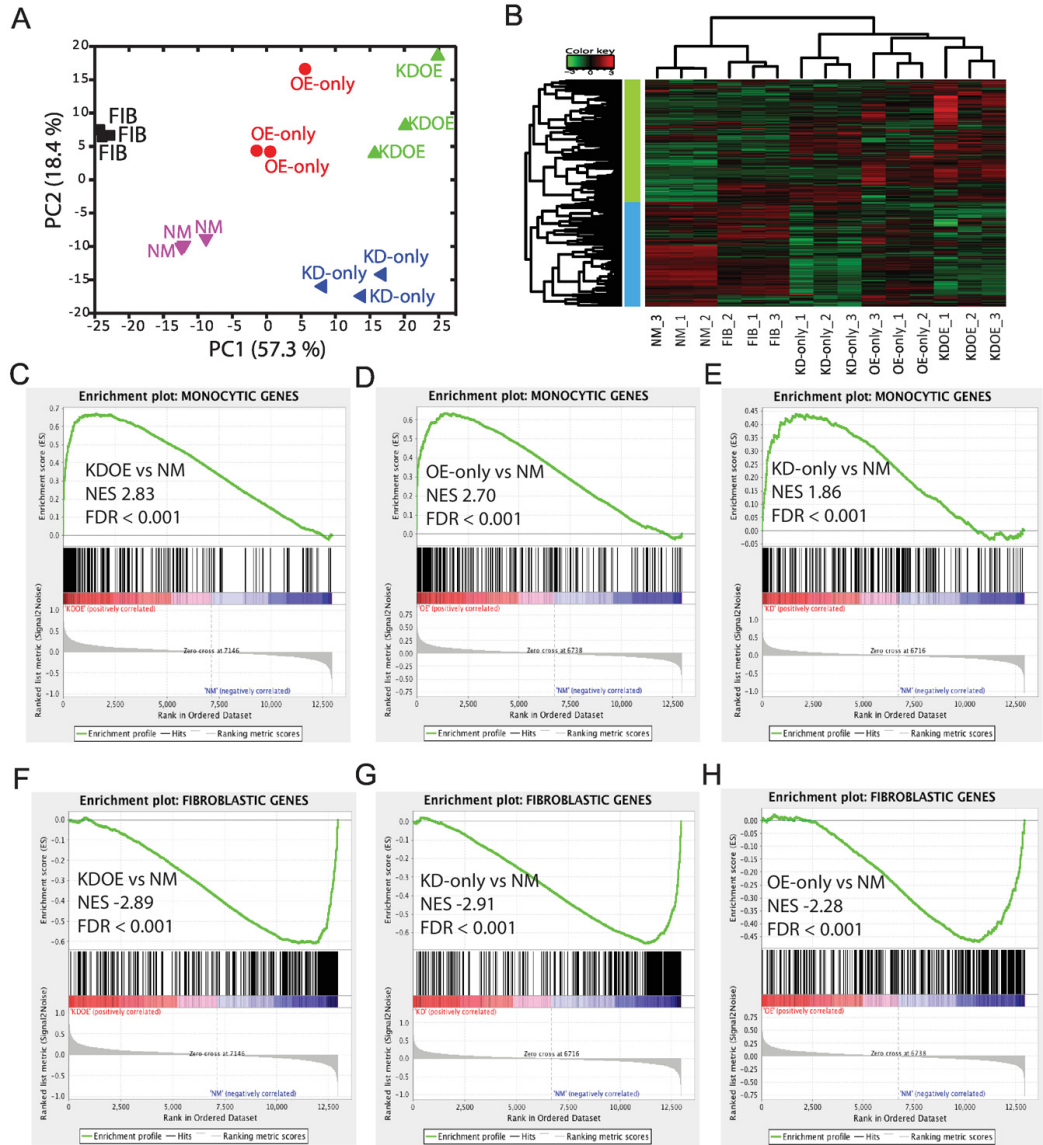
Microarray analysis of monocytic and fibroblastic gene expression in treated cells. (A) Principal component analysis of monocyte- and fibroblast-specific gene expression. (B) Heatmap diagram of monocyte- and fibroblast-specific gene expression. (C–E) The enrichment plot in the monocytic gene set of KDOE, OE-only, and KD-only. (F–H) Gene set enrichment analysis in the fibroblastic gene set of KDOE, KD-only, and OE-only.

Principal component analysis of all specific genes demonstrated that the component scores of KDOE were comparable to the summation of KD-only and OE-only when referred to NM, suggesting that KDOE possesses a more distinct expression pattern and enhancement occurs through the combination of fibroblastic TRN suppression and monocytic TRN induction (Fig 2A). It is clear that KD-only and OE-only were divided by the second component and deviated from NM into two different directions, revealing two different forces changing cells from the original state. Note that NM cells and fibroblasts were oppositely correlated with second component although both were separated from other three treated cells by first component, suggesting the effect of the medium change, siRNA transfection, and/or lentivirus transduction that we introduced into cells.

We then analyzed the different expression patterns by plotting a heat map. As shown in Fig 2B, even with a short incubation time, KDOE showed a reversed expression pattern compared with fibroblasts and NM; the most highly expressed fibroblastic genes and lowly expressed monocytic genes, shown in red and green in fibroblasts and NM, respectively, were suppressed and upregulated simultaneously. Interestingly, the up-regulation of monocytic genes in KDOE was higher than that of OE-only, while the downregulation of fibroblastic genes in KD-only was comparable to KDOE, revealing the enhancement of monocytic gene expression in KDOE. In support of our previous report, while overexpression of monocytic TFs partially decreased fibroblast gene expression [25], OE-only slightly downregulated fibroblastic genes. Similarly, KD-only showed upregulation of some monocytic genes, suggesting cross-regulation between two TRNs. These results were similar when we selected top 100, top 200, and top 300 specific genes expressed in monocytes and fibroblasts (S2A–S2C Fig).

Heat map analysis suggests that fibroblastic TRN may disturb monocytic gene induction and monocytic TRN may suppress fibroblastic gene expression. In order to confirm this regulation, profiles of two gene sets (381 monocyte-specific genes and 406 fibroblast-specific genes) were further subjected to the gene set enrichment analysis (GSEA) [28]. By calculating the enrichment of highly expressed genes in fibroblastic and monocytic gene sets, GSEA provides normalized enrichment score (NES), indicating how the expression profile of each treatment differs in defined gene sets, where the NES difference of 0.1 is considered to be significant. As recommended by the GSEA program, we set the stringent criteria at FDR <0.05 for significant NES. We confirmed that KD-only showed significant enrichment in monocytic genes, although the NES was relatively lower than KDOE and OE-only (Fig 2C–2E). Similar significant enrichment was confirmed in OE-only for fibroblastic genes even with lower NES (Fig 2F–2H), revealing the cross-regulation of two TRNs. Interestingly, we found that KDOE exhibited relatively higher NES than OE-only in the monocytic gene set (Fig 2C and 2D), whereas similar NES with KD-only was seen in the fibroblastic gene set (Fig 2F and 2G), suggesting the asymmetric regulation of two gene sets.

### Active interaction of two TRNs in monocytic gene regulation

**Fig 3:**
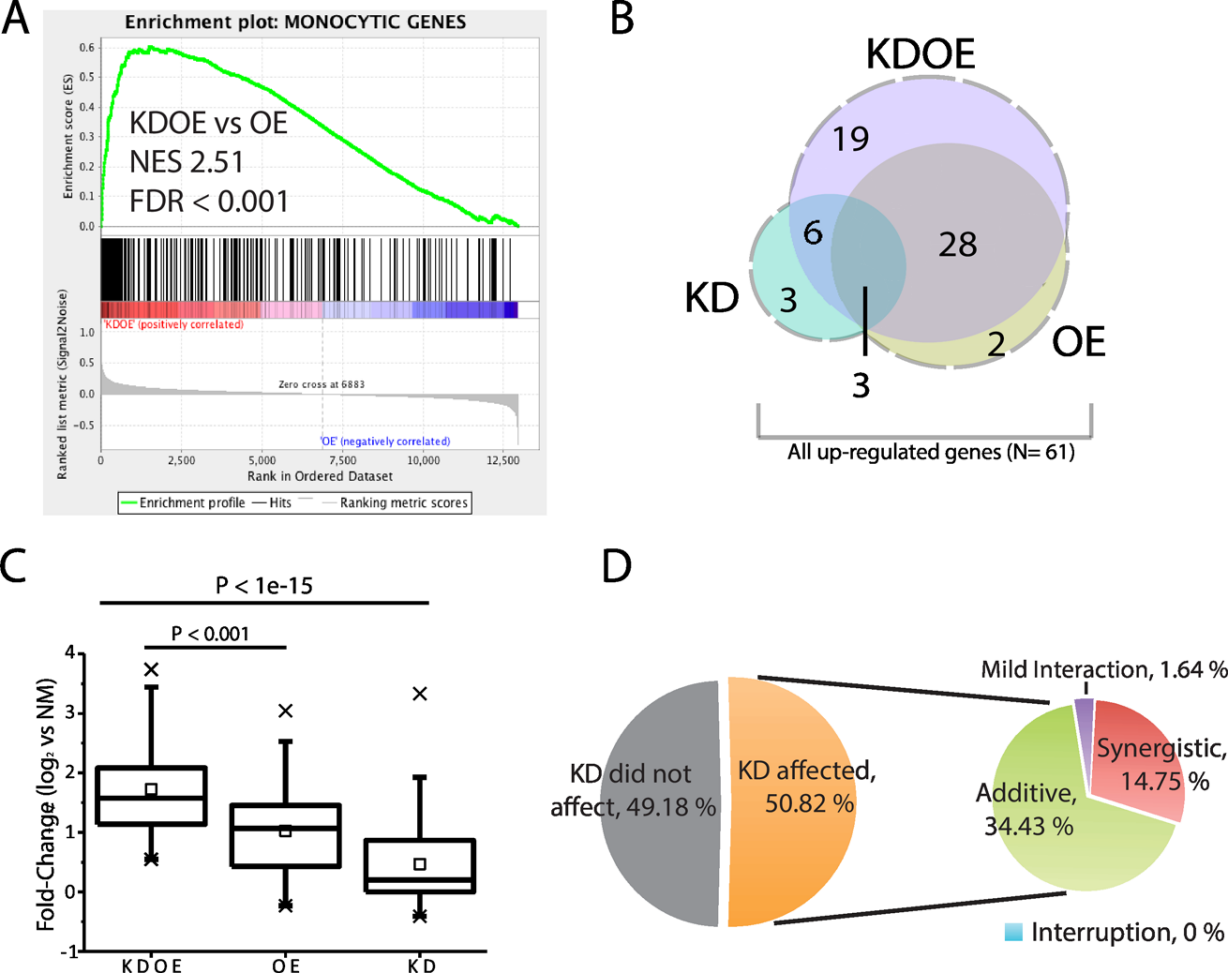
Active interaction of two TRNs in monocytic gene regulation. (A) Analysis of KDOE against OE-only shows significant enrichment. (B) Venn diagram of genes upregulated in each treatment. (C) Box-plots of fold-change distribution for all genes upregulated (N = 61, x: 1% and 99%, ☐: mean). (D) Pie charts showing the interactions between two TRNs in regulating monocytic gene expression.

To confirm the asymmetry of gene regulation, we first verified the significant enhancement of monocytic gene expression in KDOE by directly comparing the enrichment of KDOE against OE-only. As expected, Fig 3A shows KDOE revealed significant enrichment in the monocytic gene set (NES: 2.51, FDR <0.001), manifesting that monocytic gene expression was improved in KDOE. Since KD-only revealed significant enrichment in the monocytic gene set, despite the lower score (NES: 1.85, FDR <0.001, Fig 2E), we speculated that fibroblastic TRN might oppose the expression of monocytic genes. Thus, suppression of fibroblastic TRN resulted in a higher enrichment score in KDOE than OE-only.

To investigate how much monocytic gene expression is affected by two TRNs, we compared the expression among the engineered cells. Of 381 monocyte-specific genes, 56 genes (14.7%) were significantly upregulated (log_2_FC ≥1, FDR <0.05) in KDOE, although only 33 genes (8.7%) and 12 genes (3.1%) were upregulated in OE-only and KD-only, respectively (Fig 3B). The upregulated genes in OE-only highly overlapped with those in KDOE since 31 out of 33 genes were upregulated in both KDOE and OE-only. We then analyzed the fold-change distributions of all upregulated genes (N = 61, dashed line area in Fig 3B). The box plots shows that KDOE exhibited significantly greater fold-change distribution than OE-only (p <0.001, two-sample Kolmogorov-Smirnov (K-S) test, Fig 3C). The dramatically increased monocytic gene expression in KDOE again suggests the interference of fibroblastic TRN in monocytic gene regulation.

To further uncover how much fibroblastic TRN repulsed monocytic gene expression, we identified genes that were significantly affected by fibroblastic TRN suppression (i.e., KD) by comparing the fold-change of each gene between KDOE and OE-only. Surprisingly, 31 out of 61 (50.82%) upregulated monocytic genes were affected by fibroblastic TRN suppression (t-test, FDR <0.05). Importantly, all affected genes were positively affected (fold-change larger in KDOE than OE-only), showing that fibroblastic TRN prominently suppress monocytic gene expression. Moreover, we found that nine genes were significantly higher expressed in KDOE than the summation of KD-only and OE-only, revealing the synergistic effect of knocking-down and overexpression (Fig 3D). This synergistic enhancement suggests that in addition to passive repulsion, fibroblastic TRN actively disturbs monocytic TRN to inhibit these genes. Twenty-one out of 31 KD-affected genes in KDOE showed fold-changes equal to the summation of KD-only and OE-only, indicating that fibroblastic TRN suppresses but works independently with monocytic TRN on those genes (additive, 34.43%). Note that only 1 gene (1.64%) was expressed in a fold-change value in KDOE significantly higher than OE-only, although lower than summation, showing relatively mild enhancement by KD. These results are consistent with our speculation that fibroblastic TRN regulates monocytic genes and demonstrate that the regulation can involve active interactions with monocytic TRN on certain genes.

### Most fibroblastic genes are not regulated by monocytic TRNs

**Fig 4:**
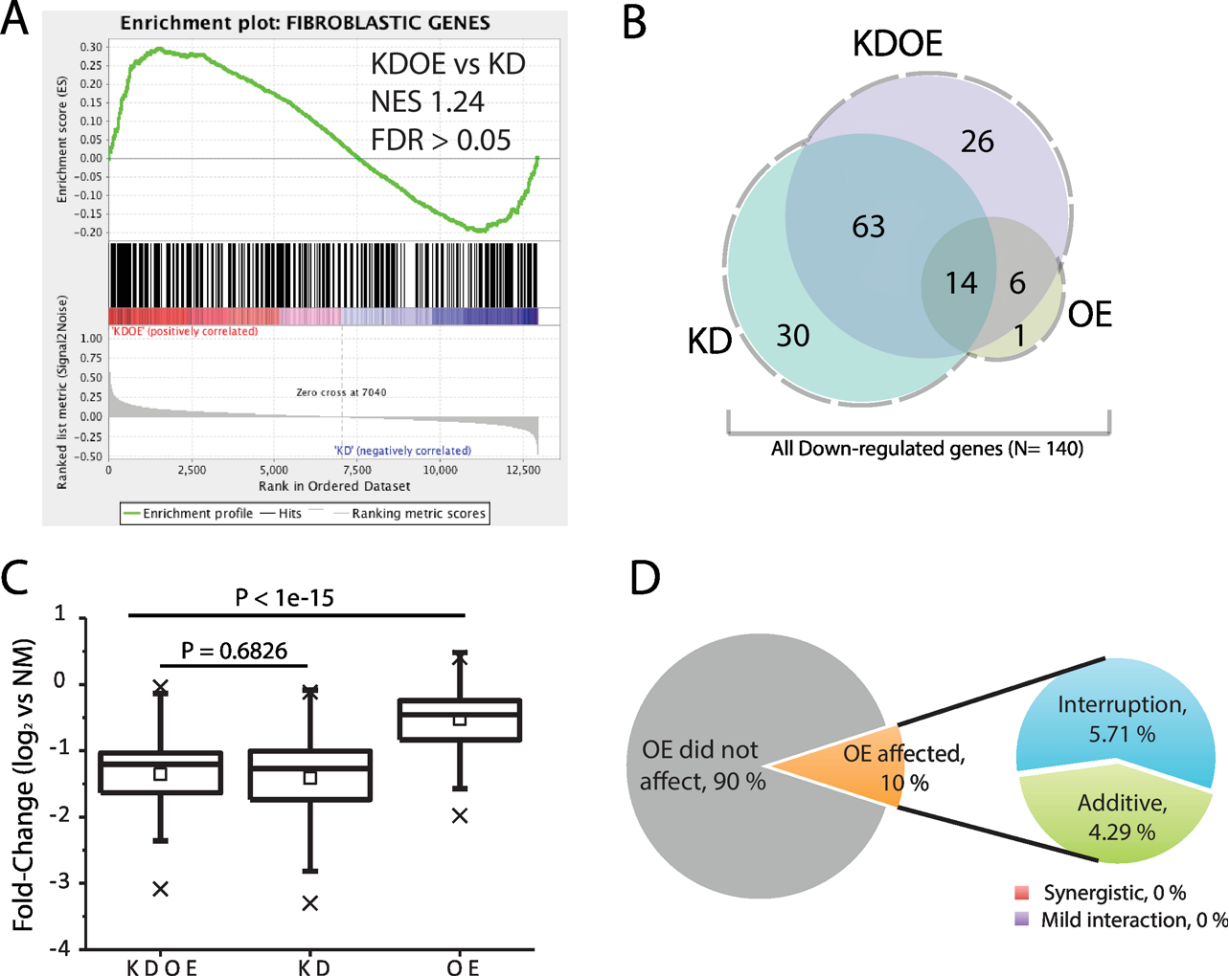
Poor regulations of monocytic TRN in fibroblastic gene suppression. (A) The enrichment was insignificant when we analyzed KDOE against KD-only. (B) Venn diagram showing overlapped gene numbers downregulated in each treatment. (C) The box-plot for fold-change distributions of all genes downregulated (N = 140, x: 1% and 99%, ☐: mean). (D) The interactions of two TRNs affecting fibroblastic gene suppression.

We further examined the poor facilitation of fibroblastic gene suppression in KDOE. In accordance with the similar enrichment scores of KDOE and KD-only in the fibroblastic gene set (Fig 2F and 2G), the enrichment of KDOE against KD-only was insignificant (NES: 1.24, FDR >0.05), indicating that in general, the suppression in KDOE was not improved from KD-only (Fig 4A).

Out of 406 fibroblastic genes, 107 (26.3%) and 109 (26.8%) were significantly downregulated (log_2_FC ≤1, FDR <0.05) in KD-only and KDOE, respectively, although only 21 (5.2%) genes were suppressed in OE-only. The Venn diagram shows that downregulated fibroblastic genes in OE-only highly overlapped with those in KDOE (20 out of 21 (95.2%) genes), although overlap of KD-only with KDOE (77 out of 107 (72.0%) genes) was intermediate (Fig 4B). Using all significantly downregulated fibroblastic genes (N = 140, dashed line area in Fig 4B), we again compared the fold-change of each sample. Unlike the significantly enhanced upregulation of monocytic genes (Fig 3C), the fold-change of fibroblastic genes in KDOE was insignificant to that of KD-only (p = 0.6826, K-S test, Fig 4C). Furthermore, fold-change analysis of each gene showed that most of the downregulated fibroblastic genes (90.0%) were not affected by monocytic TRN (t-test, FDR >0.05). In 14 OE-affected genes (10.0%), none of them showed significantly facilitated suppression in KDOE. Monocytic TRN showed additive suppressive effects on the knockdown of fibroblastic TRN in only six genes (4.29%, additive) and a surprisingly interruptive effect in eight genes (5.71%). These results demonstrate that monocytic TRN regulates few fibroblastic genes expression.

## Discussion

Cell reprogramming involves mysterious and complex processes comprising the reconstitution and deconstruction of different TRNs; the intrinsic network that safeguards the stability of starting cells may interfere with gene regulation of the induced TRN, resulting in poor reprogramming efficiency [13,14,29–31]. Here we investigated the gene regulation of intrinsic and induced TRNs by making contrast of the combinatorial treatment (KDOE) with each individual one (KD-only and OE-only). We found that knocking down fibroblastic TRN positively affects half of the upregulated monocytic genes, demonstrating that intrinsic fibroblastic TRN indeed interfere with expression of monocytic genes (Fig 3D). In addition, we found knockdown of fibroblastic TRN upregulated other monocytic TFs, which were identified previously [25] (S4A Fig), suggesting that synergistically upregulated monocytic genes might result from the active interaction of fibroblastic TRN to monocytic TRN. TFs not only upregulate the expression of downstream genes but also oppose other TFs and their downstream genes to manage cell lineage development [32–34]. Therefore, our results support the idea that in addition to passively securing the stability of intrinsic genes, innate TRN actively interfere with the expression of other lineages genes.

On the other hand, unlike the significant effect in regulating monocytic gene expression, induced monocytic TRN showed neutral effects on the downregulation of most fibroblastic genes (Fig 4D). Even in the monocytic TRN-affected fibroblastic genes, both suppressive and inducible effects were observed. However, the results should be carefully interpreted if we accept that innate TRN actively interferes with expression of other lineages genes, given that fibroblastic genes are other lineage genes from the standpoint of monocytic TRN. It is unlikely that monocytic TRN is exceptionally neutral in interfering with fibroblastic genes. We assume that the results may be explained due to uncompleted construction of monocytic TRN, which resulted from relatively shorter incubation duration for the observation. It is also possible that the suppressive effect of other lineage genes is accompanied at a later stage of induced TRN reconstitution.

Nonetheless, it is important that two TRNs asymmetrically regulate intrinsic and induced gene sets while the induced TRN is under construction in reprogramming. The significantly enhanced expression of monocytic genes by knocking-down fibroblastic TRN reminds us of the importance of the signature of innate TRN during the reprogramming process [35–37]. We previously reported that suppressing fibroblastic TRN, together with the adipocyte differentiation medium, facilitated the transdifferentiation to adipocytes [24]. Furthermore, there are several reports that overexpression target cell specific miRNA facilitated cell reprogramming [38,39], in which the induced miRNAs are considered to suppress gene expression regulated by innate TRN. Consider the fact that induced monocytic TRN exert subtle effects on suppressing fibroblastic genes, it is highly possible that knockdown of innate TRN, together with induction of target cell TRN, can be coordinated for duration shortening or quality optimization in other cell conversion experiments from fibroblast cells. Further investigation into the mechanism for these interactions will not only lead to a greater understanding of the reprogramming process but also be conducive to the important TRNs exploration.

## Supporting Information

S1 Fig Overexpression of Four monocytic TFs at a time. (A) Diagram of Plasmid construct. Four genes were connected by poly A sequence and followed by a blasticidin-resistance gene (ORF_2). (B) Confirmation of overexpression using western blot. In addition to the four cell lines we constructed mentioned in the main text, we expressed individual protein in fibroblasts and run them as markers to indicate the correct protein position. As expected, all four proteins were expressed in KDOE and OE-only, but not in NM and KD-only. All the protein samples were loaded at 1.5 ng/μL for gel electrophoresis and were stained with rabbit polyclonal antibody (abcam, England) according to the manual instruction.
(PDF)

S2 Fig **Heat map diagram of (A) top 100 genes, (B) top 200 genes, and (C) top 300 monocytic and fibroblastic genes.** These results showed similar results compared with all specific genes plotted in Fig 2B.
(PDF)

S3 Fig **Gene set enrichment analysis of OE-only against KD-only (A-B) and KDOE against KD-only/ OE-only (C-D).** (A & B) Fibroblastic genes and monocytic genes were higher expressed in OE-only than KD-only, showing that OE-only poorly suppressed fibroblastic genes while the expressed more monocytic genes. (C) KDOE were significantly enriched in monocytic genes against KD-only. (D) Enrichment in fibroblastic genes was negative when compared to OE-only, confirming the knocking down of fibroblastic TRNW in KDOE.
(PDF)

S4 Fig **Expression levels from microarray data of (A) monocytic and (B) fibroblastic transcription factors.** (A) Knocking down fibroblastic TRNW significantly up-regulated several monocytic TFs such as *LMO2*, *MAFB*, *MAF*, *NR4A2*, and *STAT5A* (**: p–value < 0.05, t-test). (B) Overexpressing monocytic TRNW merely down-regulated significantly in *MKX*.
(PDF)

S1 Table Primer sets for qPCR. (PDF)
