# Supplementary material for: Asymmetric Regulation of Peripheral Genes by Two Transcriptional Regulatory Networks
Source: PLoS One. 2016 Aug 2;11(8):e0160459. doi: 10.1371/journal.pone.0160459 (PMC4970704; doi:10.1371/journal.pone.0160459)
Supplement: S1 Fig — (A) Diagram of Plasmid construct. Four genes were connected by poly A sequence and followed by a blasticidin-resistance gene (ORF_2). (B) Confirmation of overexpression using western blot. In addition to the four cell lines we constructed mentioned in the main text, we expressed individual protein in fibroblasts and run them as markers to indicate the correct protein position. As expected, all four proteins were expressed in KDOE and OE-only, but not in NM and KD-only. All the protein samples were loaded at 1.5 ng/μL for gel electrophoresis and were stained with rabbit polyclonal antibody (abcam, England) according to the manual instruction. (PDF) [file pone.0160459.s001.pdf]

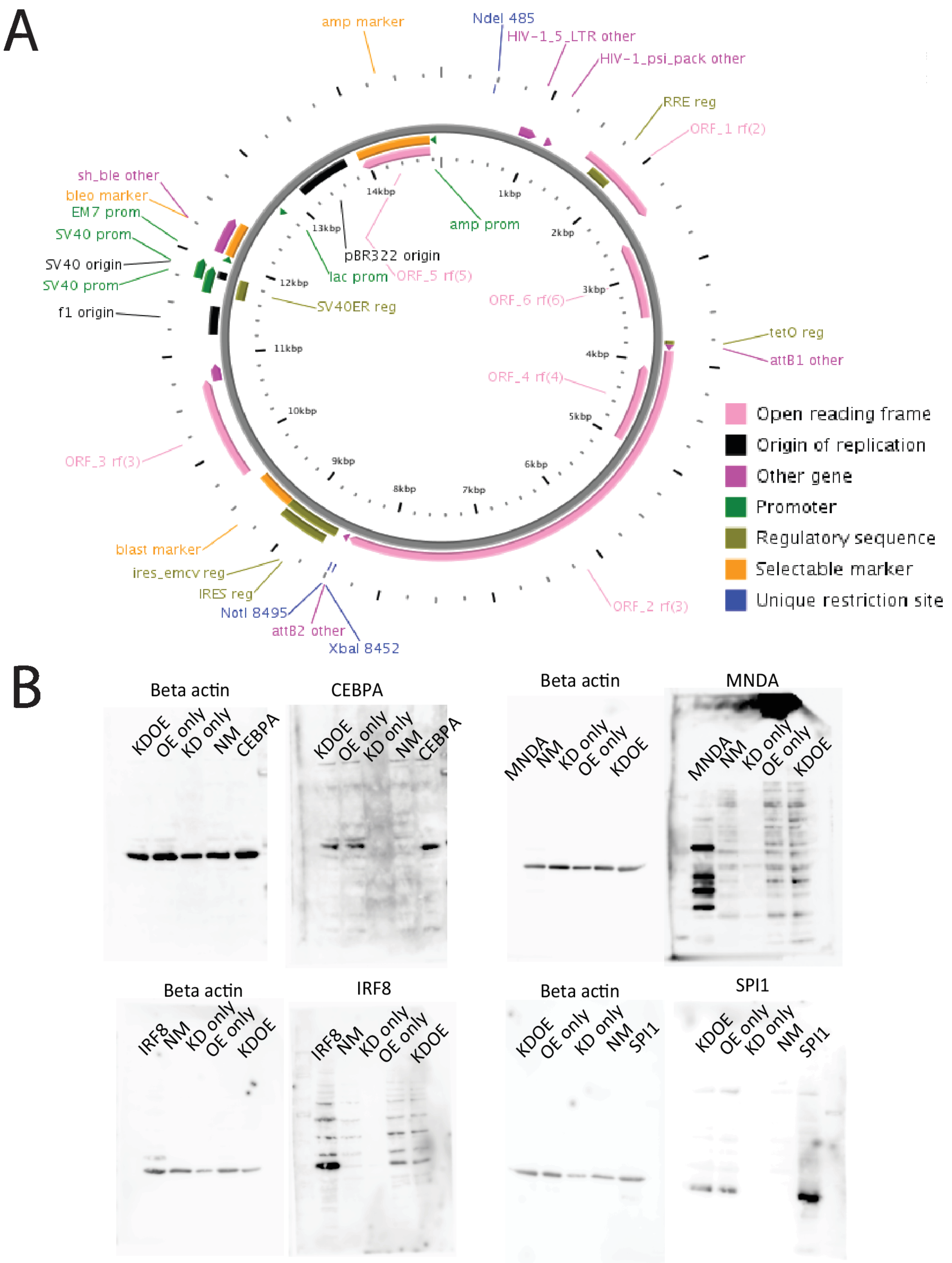


**S1 Fig. Overexpression of Four monocytic TFs at a time**. (A) Diagram of Plasmid construct. Four genes were connected by poly A sequence and followed by a blasticidin-resistance gene (ORF_2). (B) Confirmation of overexpression using western blot. In addition to the four cell lines we constructed mentioned in the main text, we expressed individual protein in fibroblasts and run them as markers to indicate the correct protein position. As expected, all four proteins were expressed in KDOE and OE-only, but not in NM and KD-only. All the protein samples were

loaded at 1.5 ng/μL for gel electrophoresis and were stained with rabbit polyclonal antibody (abcam, England) according to the manual instruction.
