# Supplementary material for: Asymmetric Regulation of Peripheral Genes by Two Transcriptional Regulatory Networks
Source: PLoS One. 2016 Aug 2;11(8):e0160459. doi: 10.1371/journal.pone.0160459 (PMC4970704; doi:10.1371/journal.pone.0160459)
Supplement: S2 Fig — Heat map diagram of (A) top 100 genes, (B) top 200 genes, and (C) top 300 monocytic and fibroblastic genes. These results showed similar results compared with all specific genes plotted in Fig 2B. (PDF) [file pone.0160459.s002.pdf]

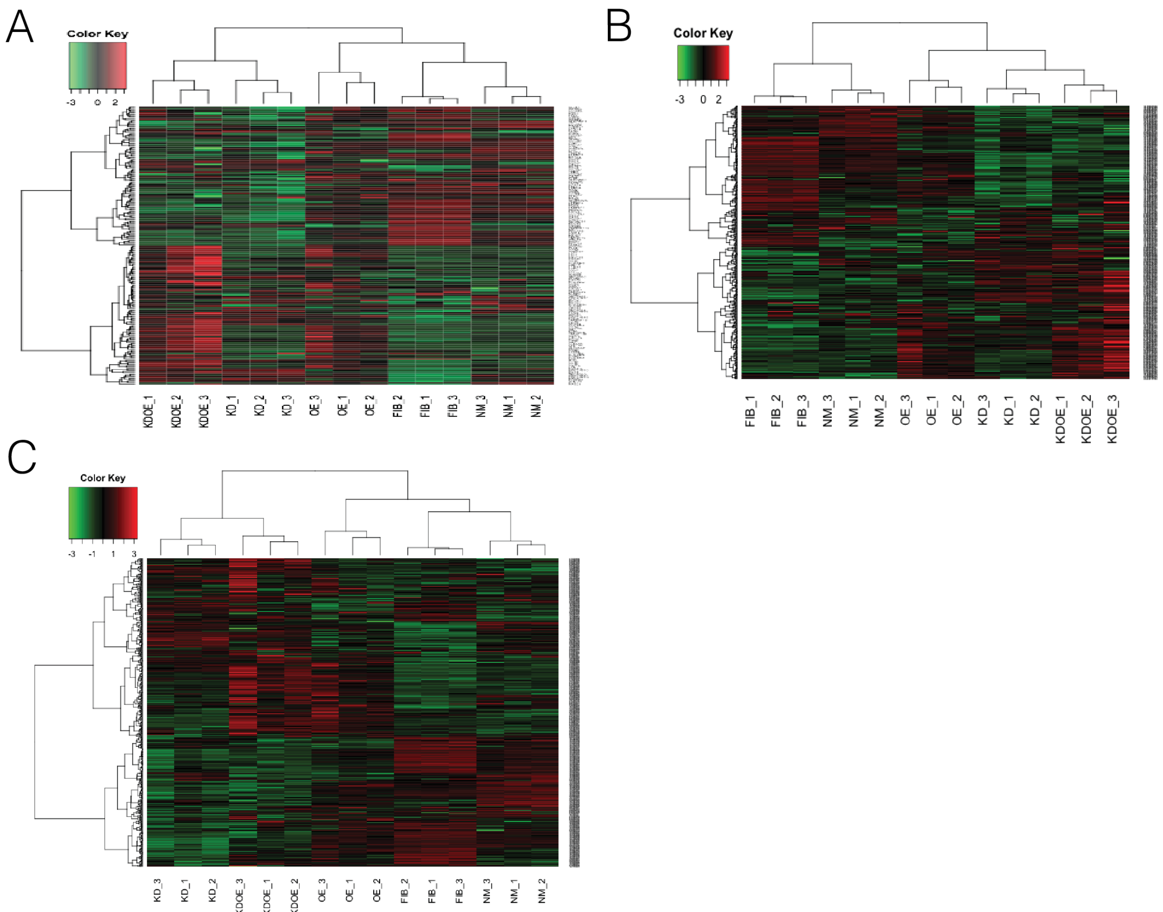

S2 Fig. Heatmap diagram of (A) top 100 genes, (B) top 200 , and (C) top 300 monocytic and fibroblastic genes. These results showed similar results compared with all specific genes plotted in Fig 2B.
