# Supplementary material for: Asymmetric Regulation of Peripheral Genes by Two Transcriptional Regulatory Networks
Source: PLoS One. 2016 Aug 2;11(8):e0160459. doi: 10.1371/journal.pone.0160459 (PMC4970704; doi:10.1371/journal.pone.0160459)
Supplement: S3 Fig — Gene set enrichment analysis of OE-only against KD-only (A-B) and KDOE against KD-only/ OE-only (C-D). (A & B) Fibroblastic genes and monocytic genes were higher expressed in OE-only than KD-only, showing that OE-only poorly suppressed fibroblastic genes while the expressed more monocytic genes. (C) KDOE were significantly enriched in monocytic genes against KD-only. (D) Enrichment in fibroblastic genes was negative when compared to OE-only, confirming the knocking down of fibroblastic TRNW in KDOE. (PDF) [file pone.0160459.s003.pdf]

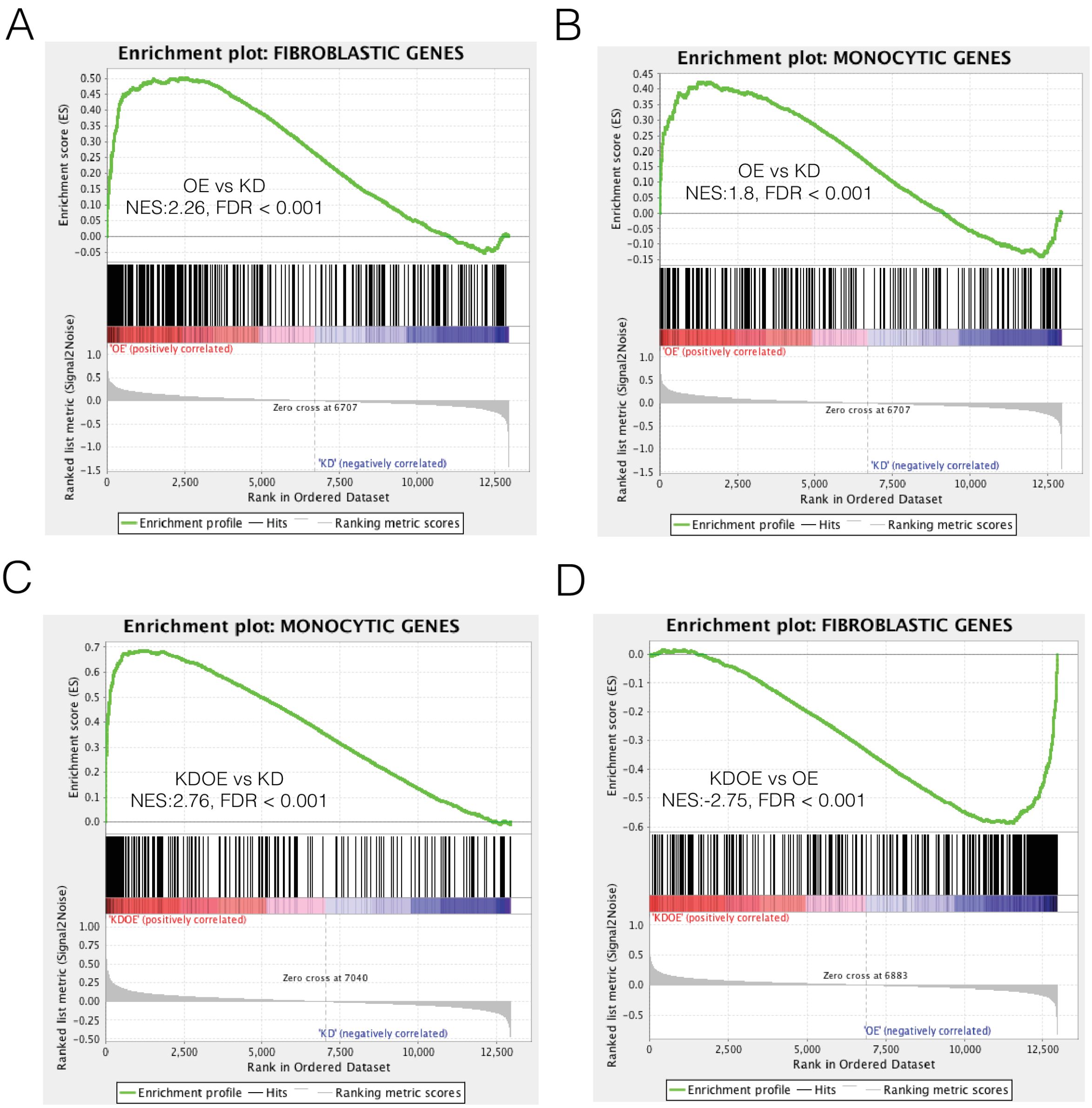

S3 Fig. Gene set enrichment analysis of OE-only against KD-only (A-B) and KDOE against KD-only/ OE-only (C-D). (A & B) Fibroblastic genes and monocytic genes were higher expressed in OE-only than KD-only, showing that OE-only poorly suppressed fibroblastic genes while the expressed more monocytic genes. (C) KDOE were significantly enriched in monocytic genes against KD-only. (D) Enrichment in fibroblastic genes was negative when compared to OE-only, confirming the knocking down of fibroblastic TRNW in KDOE.
