# Supplementary material for: Asymmetric Regulation of Peripheral Genes by Two Transcriptional Regulatory Networks
Source: PLoS One. 2016 Aug 2;11(8):e0160459. doi: 10.1371/journal.pone.0160459 (PMC4970704; doi:10.1371/journal.pone.0160459)
Supplement: S4 Fig — Expression levels from microarray data of (A) monocytic and (B) fibroblastic transcription factors. (A) Knocking down fibroblastic TRNW significantly up-regulated several monocytic TFs such as LMO2, MAFB, MAF, NR4A2, and STAT5A (**: p–value < 0.05, t-test). (B) Overexpressing monocytic TRNW merely down-regulated significantly in MKX. (PDF) [file pone.0160459.s004.pdf]

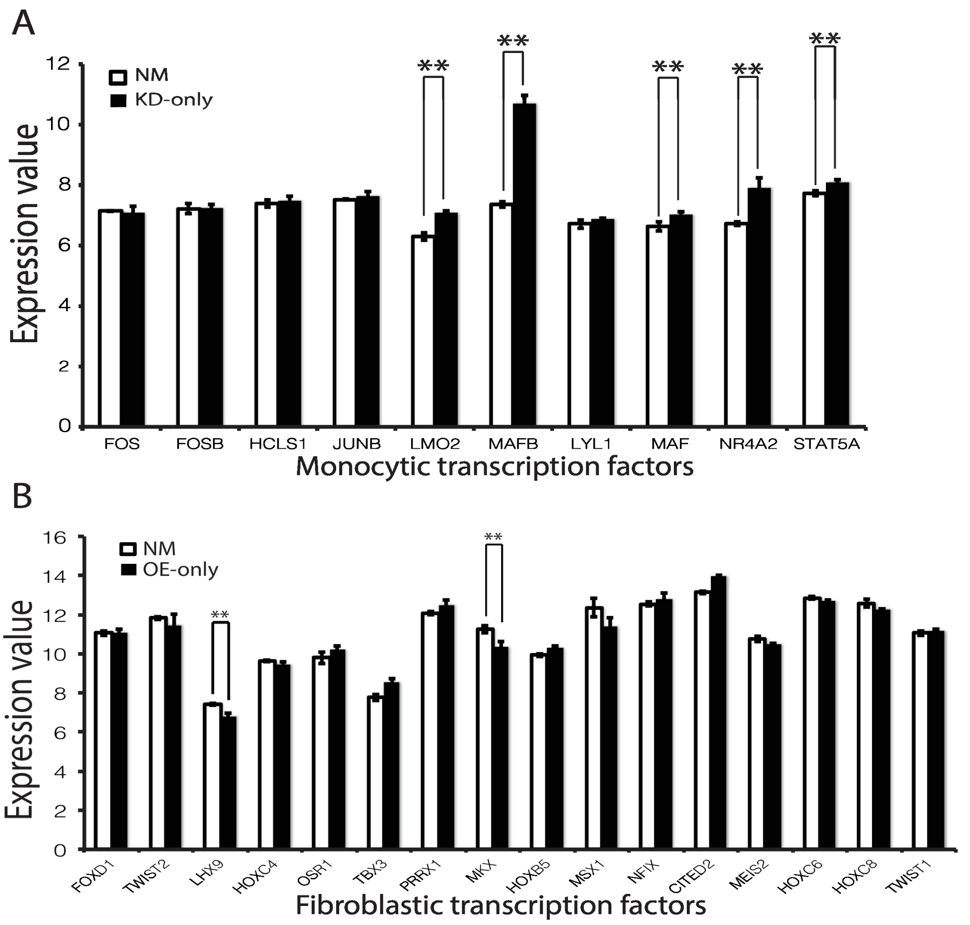

S4 Fig. Expression levels from microarray data of other monocytic (A) and fibroblastic (B) transcription factors. (A) Knocking down fibroblastic TRNW significantly up-regulated several monocytic TFs such as *LMO2*, *MAFB, MAF, NR4A2,* and *STAT5A* (**: p –value < 0.05, t-test). (B) Overexpressing monocytic TRNW merely down-regulated significantly in *MKX*.
