# Supplementary material for: Asymmetric Regulation of Peripheral Genes by Two Transcriptional Regulatory Networks
Source: PLoS One. 2016 Aug 2;11(8):e0160459. doi: 10.1371/journal.pone.0160459 (PMC4970704; doi:10.1371/journal.pone.0160459)
Supplement: S1 Table — (PDF) [file pone.0160459.s005.pdf]

S1 Table. Primers sets for qPCR

**(A) Fibroblastic TFs**	
OSR1_Fw	AAGACCACCTGCGAGACCA
OSR1_Rv	CTTGTGGACAGCGAGAGTCC
PRRX1_Fw	GAGACCCACCGATTATCTCTCC
PRRX1_Rv	CGTTATGAAGCCCCTCGTGT
LHX9_Fw	GCTTGCCCAGAAAACAGGTC
LHX9_Rv	TCCTGCCGCAAAAGGTTC
TWIST2_Fw	GAGCAAGATCCAGACGCTCA
TWIST2_Rv	TGGTCATCTTATTGTCCATCTCGT

**(B) Monocytic TFs**	
SPI1_Fw	CAAAGATAAGGGCACATTCCAGTT
SPI1_Rv	GGTCATCTTCTTCCTATTTCCCTTC
CEBPA_Fw	CAGCATTGACATTTCCGCTTAC
CEBPA_Rv	TGCTTTTGCCTTTTCCTGCT
MMDA_Fw	GGCGTGATGGAGATAAAAGAGG
MNDA_Rv	GGTGTCTTGTTTGCGATTTCTATG
IRF8_Fw	ACGGCAGGGAGTGTTTGTG
IRF8_Rv	CGTTCCAGCTTGTTGGGTCT

**(C) LPS stimulated genes**	
TNF_Fw	GCCTGCTGCACTTTGGAGT
TNF_Rv	CTCGGGGTTCGAGAAGATG
IL6_Fw	CACCTCTTCAGAACGAATTGACA
IL6_Rv	CCTCTTTGCTGCTTTCACACA
IL1B_Fw	TTTGAAGCTGATGGCCCTAAA
IL1B_Rv	GTGGTGGTCGGAGATTCGTAG
CCL2_Fw	CAGCAGCAAGTGTCCCAAA
CCL2_Rv	ATGGAATCCTGAACCCACTTCT
CXCL10_Fw	TTCCTGCAAGCCAATTTTGT
CXCL10_Rv	TGATGGCCTTCGATTCTGG
IFNB1_Fw	ATCTAGCACTGGCTGGAATGAG
IFNB1_Rv	CCAGGACTGTCTTCAGATGGTTT
CCL3_Fw	CAGATTCCACAGAATTTCATAGCTG
CCL3_Rv	CGGCTTCGCTTGGTTAGGA
